# Formation and development of the male copulatory organ in the spider *Parasteatoda tepidariorum* involves a metamorphosis-like process

**DOI:** 10.1038/s41598-019-43192-9

**Published:** 2019-05-06

**Authors:** Felix Simon Christian Quade, Jana Holtzheimer, Jasper Frohn, Mareike Töpperwien, Tim Salditt, Nikola-Michael Prpic

**Affiliations:** 1 Georg-August-Universität Göttingen, Johann-Friedrich-Blumenbach-Institut für Zoologie und Anthropologie, Abteilung für Entwicklungsbiologie, Justus-von-Liebig-Weg 11, 37077 Göttingen, Germany; 2 Göttingen Center for Molecular Biosciences (GZMB), Ernst-Caspari-Haus, Justus-von-Liebig-Weg 11, 37077 Göttingen, Germany; 3 Georg-August-Universität Göttingen, Institut für Röntgenphysik, Friedrich-Hund-Platz 1, 37077 Göttingen, Germany; 4 Justus-Liebig-Universität Gießen, Allgemeine Zoologie und Entwicklungsbiologie, Carl-Vogt-Haus, Heinrich-Buff-Ring 38, 35392 Gießen, Germany

**Keywords:** Zoology, Organogenesis

## Abstract

Spiders have evolved a unique male copulatory organ, the pedipalp bulb. The morphology of the bulb is species specific and plays an important role in species recognition and prezygotic reproductive isolation. Despite its importance for spider biodiversity, the mechanisms that control bulb development are virtually unknown. We have used confocal laser scanning microscopy (CLSM) and diffusible iodine-based contrast-enhanced micro computed tomography (dice-µCT) to study bulb development in the spider *Parasteatoda tepidariorum*. These imaging technologies enabled us to study bulb development *in situ*, without the use of destructive procedures for the first time. We show here that the inflated pedipalp tip in the subadult stage is filled with haemolymph that rapidly coagulates. Coagulation indicates histolytic processes that disintegrate tibia and tarsus, similar to histolytic processes during metamorphosis in holometabolous insects. The coagulated material contains cell inclusions that likely represent the cell source for the re-establishment of tarsus and tibia after histolysis, comparable to the histoblasts in insect metamorphosis. The shape of the coagulated mass prefigures the shape of the adult tarsus (cymbium) like a blueprint for the histoblasts. This suggests a unique role for controlled coagulation after histolysis in the metamorphosis-like morphogenesis of the male pedipalp.

## Introduction

With over 1 million described species, arthropods are by far the most speciose group of animals^1^. The arthropods comprise four major groups: Insecta (e.g. beetles, butterflies), Chelicerata (e.g. spiders, mites, scorpions), Myriapoda (e.g. millipeds, centipeds), and Crustacea (e.g. crabs, shrimp). Originally the arthropods are an aquatic (marine) group, but in all four major groups one or several events of terrestrialisation have occurred^2,3^. The conquest of land requires not only the evolution of novel strategies for breathing in air, but also new ways for the safe transfer of sperm from the male to the female. In the aquatic environment, the gametes can simply be released into the water. Under terrestrial conditions a more direct way of gamete delivery is required that also protects the sperm from dehydration. For this purpose, true spiders (Araneae) have evolved a unique copulatory organ in the male, the so-called pedipalp bulb. The pedipalps are a pair of segmented appendages in front of the four pairs of walking legs. In both sexes they serve for sensory perception and feeding, but in males the pedipalps are additionally modified to serve as an intromittent organ. The pedipalps are generally similar to the walking legs, but are shorter and have only six segments, instead of seven in the walking legs (reviewed by Pechmann *et al*.^4^). The last segment of the male pedipalp is scoop-shaped and is termed the cymbium. In the cymbium the bulb lies, which is used by the male to take up its own sperm and safely store it until copulation. During copulation the male uses the pedipalp to reach out for the female genital opening, insert the bulb into it, and then release the stored seminal fluid into the female genital tract. In its simplest form the bulb is a soft, sac-shaped protrusion, but in the majority of species the bulb is additionally equipped with a complex set of strongly sclerotised sclerites^5–7^. In these species, the bulb is not only used for sperm storage and transfer, but the sclerites also ensure a safe locking of the bulb inside of the female genital system to prevent a premature separation and thus sperm loss. The soft portion of the bulb, the so called haematodocha, is inflatable hydraulically (comparable to a balloon). This enables the movements of the sclerites and also provides the neccessary flexibility for the entire structure to enter the female genital opening^8,9^. The individual sclerites serve distinct functions during copulation. The ring-shaped tegulum and subtegulum stabilise the soft portions of the bulb and prevent overexpansion. The conductor makes contact to the female body and guides another sclerite, the embolus, into the genital opening. The embolus has at its tip the opening of the blind sperm duct, in which the seminal fluid is stored. The bulbs of some spider groups bear additional sclerites, the so-called apophyses, that play a role in the secure locking of the bulb in the female genitalia^10^.

All soft and sclerotised parts of the bulb together form a functional unit, which can be considered as a “key” that is adapted to fit inside the female genital opening (that can be considered as a “lock”)^5^. Thus, the bulb of a male spider fits only into the genital opening of a female of the same species. As a consequence, the shape of all bulb components is species specific and, in addition to mate choice and mating behaviour, plays an important role in species recognition and prezygotic reproductive isolation. Accordingly, the bulb is not only crucial for reproduction in spiders, its function in mate recognition also links the bulb directly to gene flow in a population and the process of speciation in spiders. An understanding of the evolutionary changes of its morphology will provide unique insight into the mechanisms of morphological evolution, especially in co-evolution with the morphology of the female genital system.

The species-specific morphology of an organ or structure is always the result of developmental processes that are in turn controlled by molecular genetic mechanisms. Thus, the evolution of the underlying molecular developmental mechanisms is the basis for morphological evolution. However, very little is known about the development of the male pedipalp and its bulb^11^, and the causal molecular and genetic mechanisms are entirely unclear. We have therefore studied the formation and further postembryonic development of the pedipalp bulb in the spider *Parasteatoda tepidariorum*, a member of the cob-web spiders (family Theridiidae). Because the morphogenesis of the bulb takes place hidden below the cuticle of the pedipalp tip, it has previously been studied only in dissected or sectioned material. In the present study we have used confocal laser scanning microscopy (CLSM) and diffusible iodine-based contrast-enhanced micro computed tomography (dice-µCT) to study bulb development in *P. tepidariorum*. These imaging technologies enabled us to visualise the forming bulb primordium *in situ*, and to describe its morphogenesis during postembryonic development of *P. tepidariorum* without the use of destructive procedures.

## Results

### General sequence and duration of the development of the pedipalp bulb

**Figure 1:**
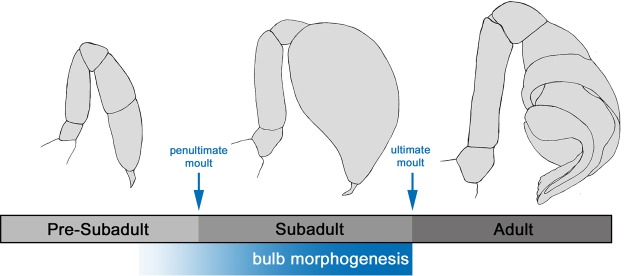
Overview of terminology used in this work. The last two moults subdivide the development of the male pedipalp into a pre-subadult, subadult, and adult stage. Bulb morphogenesis begins at the end of the pre-subadult stage and continues throughout the subadult phase. The drawings depict the typical appearance of the male pedipalp during the three stages.

The postembryonic developmental processes studied in this work take place in the penultimate (“pre-subadult”) and the ultimate nymphal instar (“subadult”; see overview in Fig. 1). The primordium of the male pedipalp bulb is first established in the pre-subadult stage (see below), but the majority of developmental processes occur during the subadult stage, i.e. between the penultimate moult and the ultimate moult. In order to measure the duration of the subadult stage we studied altogether 55 individuals that were collected at the pre-subadult stage, were provisionally sexed, and then monitored closely to record the penultimate and ultimate moults (see overview in Supplementary Table S1). The sexing of pre-subadult animals proved difficult, because at this stage external morphological differences between males and females are minimal. However, pre-subadult males show a subtle thickening of the two distal pedipalp segments, and thus could be identified with an error rate of around 10% (similar to the results by Mahmoudi *et al*.^12^). Only those individuals that were assumed to be males at the pre-subadult stage, and were confirmed to be males after the penultimate moult were accounted for, and the dates of their penultimate and ultimate moult were recorded. This resulted in a dataset of altogether 24 males with recorded dates for penultimate and ultimate moult (see Supplementary Table S1). The duration of the subadult stage varied between 10 and 17 days, with an average duration of 12.29 days. Given the fact that all individuals were kept in an incubator with controlled temperature and humidity, and were treated according to the same feeding and watering protocol (Supplementary Table S2), this variation strongly suggests intrinsic differences in developmental duration reflecting genetic diversity in developmental speed. However, we cannot entirely exclude the possibility that the differences in developmental duration were caused by external influences, because we also logged temperature and humidity within the incubator, and this revealed that despite the settings on the incubator, temperature showed a slight circadian oscillation (Supplementary Fig. S1) and humidity also undulated more strongly (between 27% and 45%) and irregularly over time (Supplementary Fig. S2).

### Origin of the bulb primordium during the pre-subadult stage

**Figure 2:**
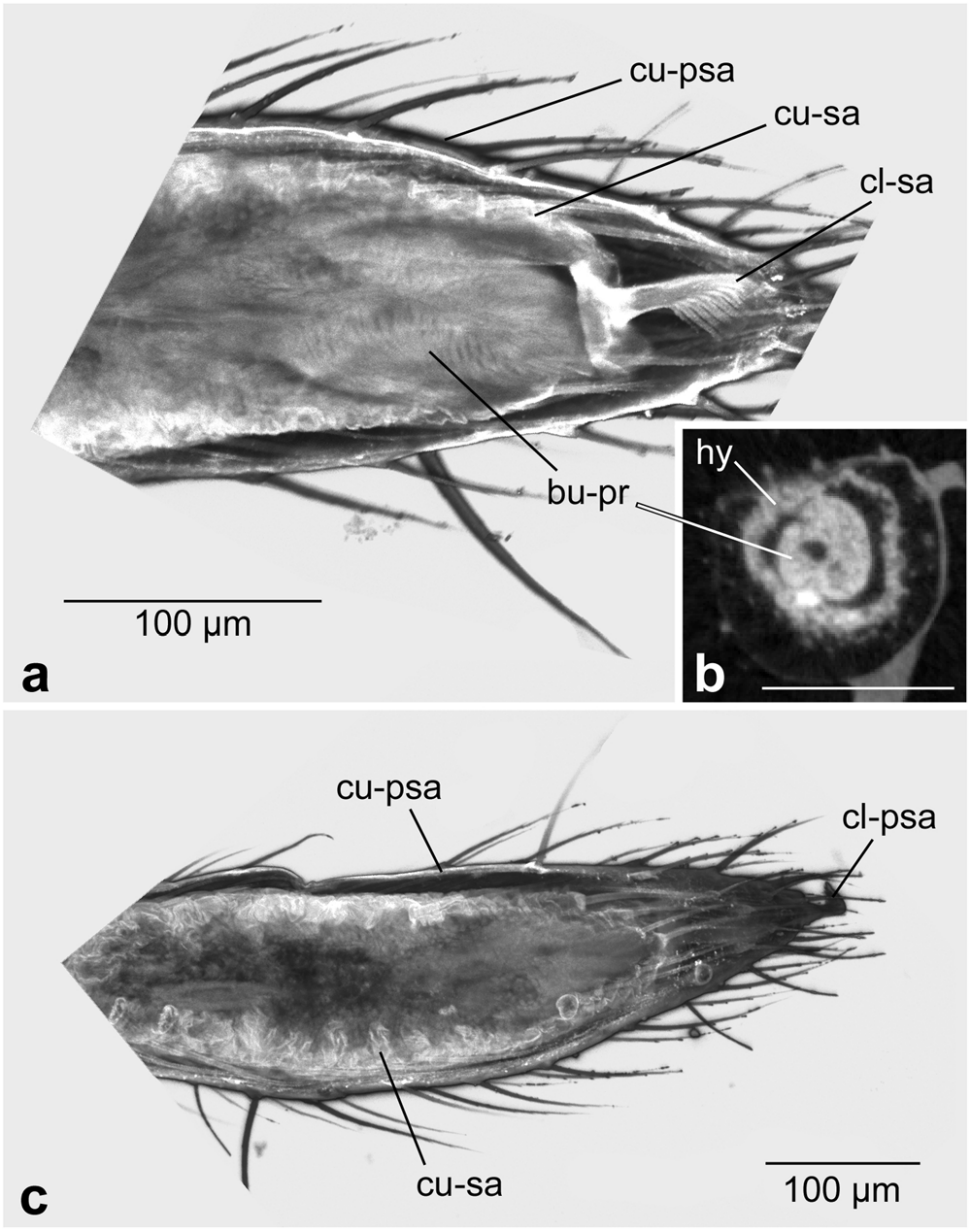
Formation of the bulb primordium at the pre-subadult stage. (**a**) Confocal LSM scan of the pedipalp tip. Distal is to the right. The ovoid primordium abuts the claw of the subadult cuticle that has already formed below the cuticle of the pre-subadult. (**b**) dice-µCT scan showing a cross-section roughly at the middle of the ovoid primordium. The primordium is internalised and surrounded by hypodermis. (**c**) Confocal LSM scan of the tarsus, distal to the right. The focal plane is set so as to show the wrinkled cuticle of the subadult beneath the smooth cuticle of the pre-subadult. Scale bar in all panels: 100 µm. Abbreviations: bu-pr, bulb primordium; cl-psa, claw of the pre-subadult; cl-sa, claw of the subadult; cu-psa, cuticle of the pre-subadult; cu-sa, cuticle of the subadult; hy, hypodermis.

The first external sign of bulb formation is a slight thickening of the two distal segments (tibia and tarsus) of the male pedipalp towards the end of the pre-subadult stage. This minor thickening is caused by the ovoid bulb primordium inside the tarsal segment, and by the formation of wrinkled subadult cuticle (see description below) underneath the cuticle of the pre-subadult. At this time point the bulb primordium is a tiny (ca. 100–150 µm long), oval organ located at the tip of the pedipalp tarsus (Fig. 2a). It is located within the tarsal tip surrounded by the tarsal hypodermis (Fig. 2b), except for the distal end which is located directly beneath the basal plate of the claw of the subadult cuticle (Fig. 2a). Thus, the cells at the tip of the bulb primordium produce the claw of the next instar, whereas the hypodermis surrounding the remainder of the primordium produces the tarsal cuticle of the next instar.

The cuticle of the pre-subadult instar surrounds the entire appendage and is close-fitting the tarsal segment. On the outside it bears a large number of bristles and the claw at its tip. The cuticle of the subadult instar that is formed beneath this pre-subadult cuticle is similar to the outer pre-subadult cuticle in bearing a large number of bristles and a distal claw. Intriguingly, however, the subadult cuticle at this stage is strongly wrinkled and folded (Fig. 2c). This surface enlargement of the subadult cuticle underneath the pre-subadult cuticle before the penultimate moult is essential for the tremendous and sudden increase of the volume of the distal portion of the male pedipalp after the penultimate moult (see next chapter).

### Morphogenesis of the bulb during the subadult stage

**Figure 3:**
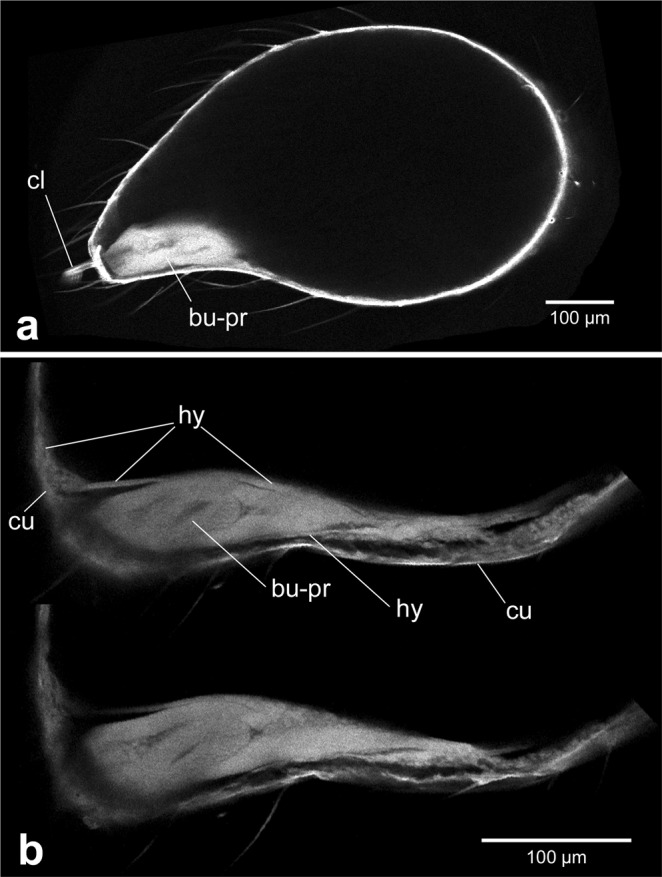
Inflation of the club at the beginning of the subadult stage. (**a**) Overview of the entire club. Confocal LSM scan. The bulb primordium occupies only a small portion within the club, the remaining club is inflated with haemolymph and this straightens out the previously wrinkled subadult cuticle. (**b**) Two different focal planes of CLSM scan showing details of bulb primordium morphology. Distal is to the left in all panels. Scale bar: 100 µm in all panels. Abbreviations: bu-pr, bulb primordium; cl, claw; cu, cuticle; hy, hypodermis.

**Figure 4:**
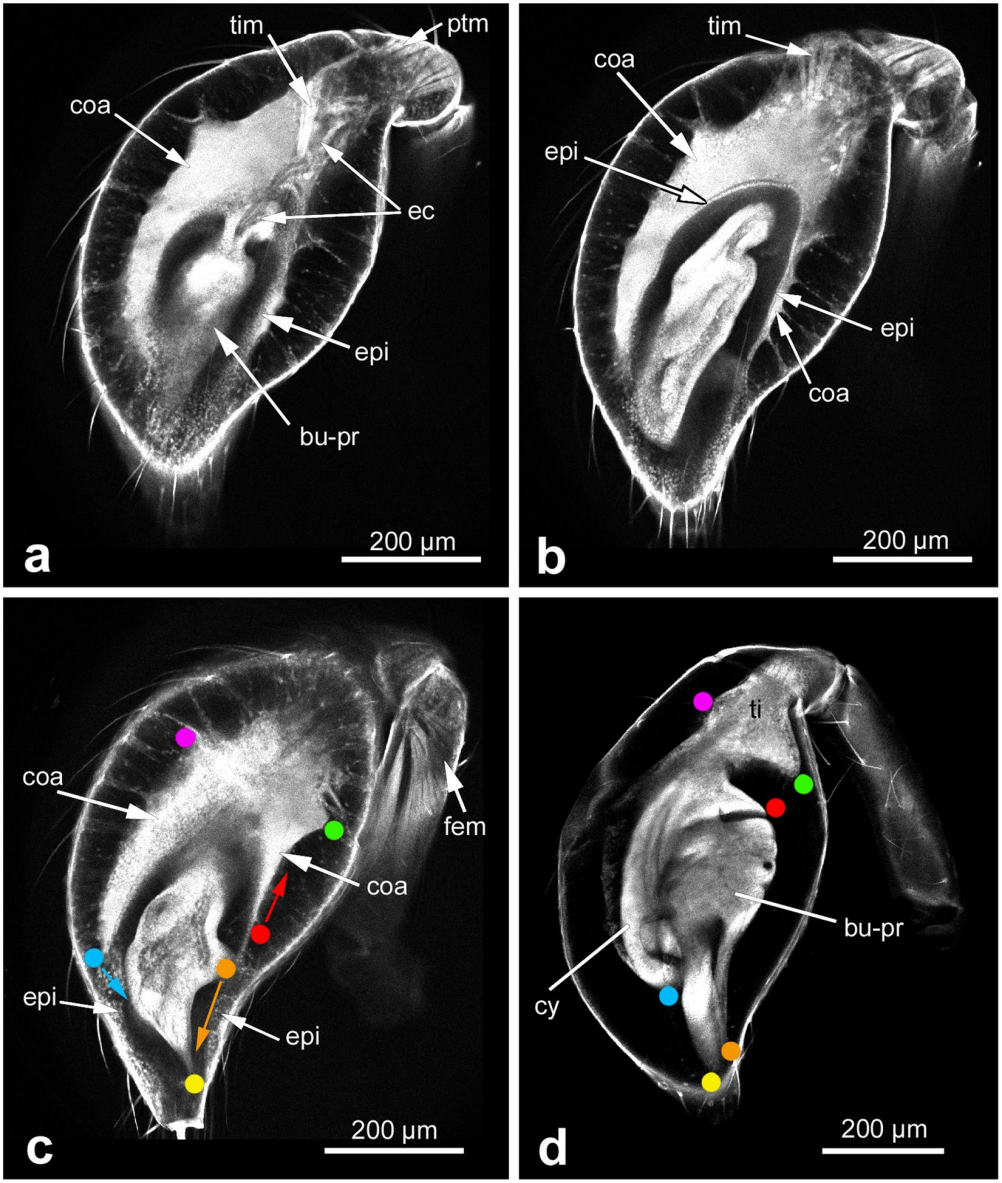
Morphogenesis of the bulb primordium at mid subadult stage. (**a**–**c**) Different focal planes of the same CLSM scan to show morphological details inside of the club. The club includes two segments, tibia and tarsus. The primordium undergoes further differentiation and a structure of coagulated material is evident. (**d**) Club shortly before the end of the subadult stage. Important landmarks that help to understand morphogenetic movements between c and d are denoted by coloured dots. The most significant movements are indicated by coloured arrows in (**c**): the tip of the future cymbium rotates towards the bulb primordium (blue arrow), the ventral rim of the future haematodocha retracts proximally (red arrow), whereas the tip of the lateral protrusion (the future embolus) rotates and shifts distally to nestle to the future conductor (orange arrow). Scale bar: 200 µm in all panels. Abbreviations: bu-pr, bulb primordium; coa, coagulated material; cy, cymbium; ec, epidermal connection; epi epithelial hypodermis; fem, femoral muscles; mes, mesenchymal hypodermis; ptm, patellar muscles; ti, tibia; tim, tibial muscle.

**Figure 5:**
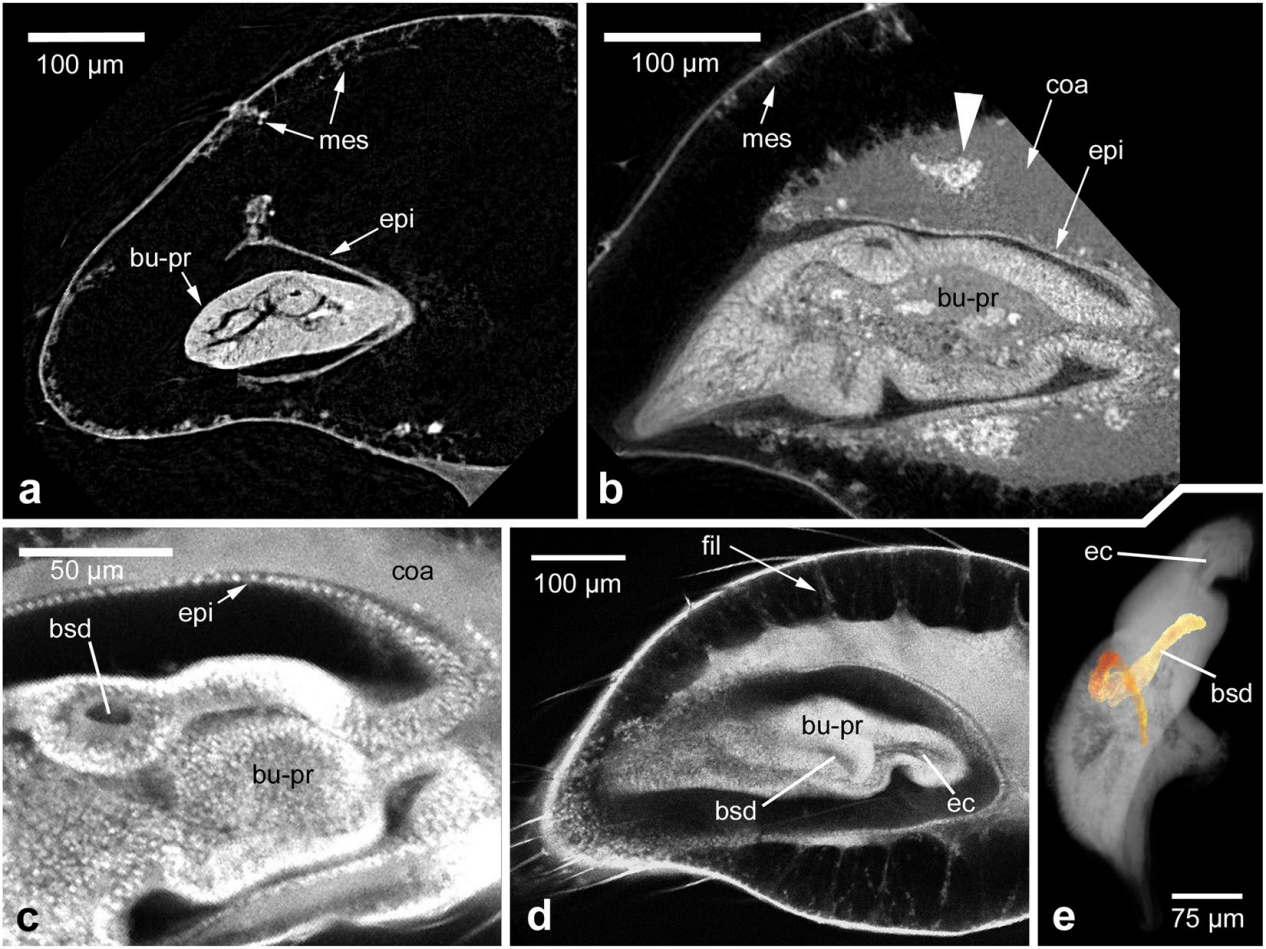
Morphological details within the developing pedipalp and bulb primordium. (**a**) Early subadult. dice-µCT scan. Distal is to the left in all panels except panel e (proximal up). The hypodermis is epithelial near the bulb primordium, but mesenchymal along the cuticle. (**b**) Mid subadult. dice-µCT scan. Coagulated material contains inclusions (arrowhead) that likely represent histoblast groups. (**c**–**e**) Details of the bulb primordium at mid subadult stage, CLSM scans. (**c**) Detail of the base of the bulb primordium, showing the epithelial lining below the coagulated material. A part of the blind sperm duct is visible in cross section. (**d**) Filaments are present between the body of the coagulated material and the mesenchymal hypodermis below the cuticle. The blind sperm duct is seen in sagittal section and a portion of the epidermal connection is visible (that leads through to the tibia, not included in this focal plane). (**e**) 3D reconstruction of the blind sperm duct within the bulb primordium (coloured in orange). The other cavity that is visible is a portion of the epidermal connection to the tibia. Scale bars: 100 µm in a, b, d; 50 µm in c; 75 µm in e. Abbreviations: bsd, blind sperm duct; bu-pr, bulb primordium; coa, coagulated material; ec, epidermal connection; fil, filaments; epi, epithelial hypodermis; mes, mesenchymal hypodermis.

After the penultimate moult the male pedipalp shows a strongly swollen distal end (the so-called “club”). As described above, the subadult cuticle is already prepared for this increase of volume at the pre-subadult stage, because it is strongly wrinkled beneath the pre-subadult cuticle. When the pre-subadult cuticle is shed, the subadult cuticle is inflated by haemolymph pressure and is straightened around the inflated distal end of the pedipalp (Fig. 3a). Most of the interior of the pedipalp club is filled with haemolymph, only at the tip of the club, directly below the claw is the primordium of the bulb, which has not significantly changed in shape or size during the moult between the pre-subadult and the subadult stages (Fig. 3a). The bulb primordium is still surrounded by the tarsal hypodermis (Fig. 3b), but sticks out at the distal end where it abuts the basal plate of the claw (Fig. 3a,b). Due to the inflation of the distal portion of the male pedipalp the hypodermis is also stretched and lines the inflated cuticle along the inside of the club. Near the bulb primordium the hypodermis forms a well-ordered epithelium (Fig. 3b, “hy”), but along the inflated cuticle the organisation of the hypodermis is less clear. In the LSM scans (that predominantly show cuticularized material due to its autofluorescence), the soft hypodermis along the inflated cuticle is hardly visible (Figs 3 and 4). However, in dice-µCT scans, that are able to better represent soft tissue, it can be seen that much of the hypodermis lines the cuticle in a mesenchymal fashion (Fig. 5a,b, “mes”), indicating the partial disintegration (histolysis) of the hypodermis.

The club is not only a swelling of the tarsus alone, but actually includes two limb segments, the tarsus and the tibia. During further development, it can be seen that the bulb primordium is connected to the patella segment through a long epidermal connection (Fig. 4a, “ec”), and also a bundle of tibial muscles is visible (Fig. 4a,b, “tim”). Inside of the epidermal connection there is a tube-like structure that is likely a primordium of blood vessels and/or nerve tracts. Another internal structure of the bulb primordium is the primordium of the blind sperm duct. In the adult, this duct opens at the tip of the embolus and serves to take up and hold the sperm until copulation. At the subadult stage, however, this duct is still completely closed and shows as a round cavity in cross section (Fig. 5c) or as an elongated cavity in sagittal section (Fig. 5d). A 3D reconstruction of the entire blind sperm duct at the mid subadult stage shows that it has a loop shape (Fig. 5e), thus prefiguring the multiple turn coil it will become in the adult.

**Figure 6:**
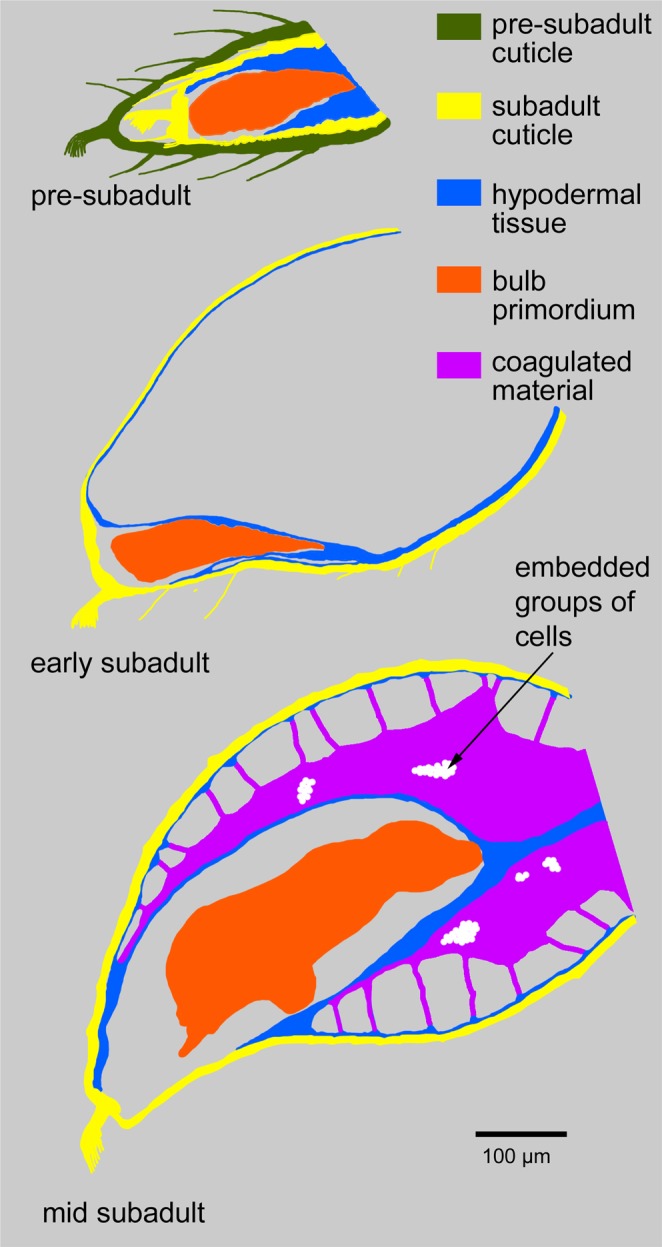
Schematic overview of the organisation of the distal end of the male pedipalp at the pre-subadult, early and mid subadult. Colours are explained in the figure. Scale bar: 100 µm.

The haemolymph filled space between the epithelially organised hypodermis near the bulb primordium and the mesenchymally organised hypodermis along most of the cuticle (Fig. 5b,c, “mes”, “epi”) increasingly fills with a fine-granular coagulated material that originates from the haemolymph in this cavity (Fig. 4a–c, Fig. 5b, “coa”). Between the mesenchymal hypodermis and the external side of the coagulated material, a large number of filaments occur that appear to tether and stabilise the forming pedipalp tip within the inflated cuticle (Figs 4a–c and 5d). The nature of these filaments is currently unclear. Although they show clearly in LSM scans, they are poorly represented in our dice-µCT scans and therefore are electron-deficient structures after iodine staining. Cells are particularly electron-rich after iodine staining, and this is known to visualise the cellular portion of a wide range of tissue types (e.g. epithelia, muscles, glands, fat tissue, nerves^13^). Therefore, the filaments represent non-cellular structures, likely also from haemolymph coagulates. The coagulated material contains dispersed inclusions that show best in dice-µCT scans (e.g. arrowhead in Fig. 5b). These structures are electron-rich and this identifies them as cell groups embedded in the matrix of the coagulated material, similar to histoblasts or groups of histoblasts (“histoblast nests”). Figure 6 gives a summary of the main components of the developing pedipalp tip and their fate during postembryonic development. In the pre-subadult, the bulb primordium fills the distal end of the tarsal segment and is partially surrounded by epithelial hypodermis. The entire appendage is surrounded by the pre-subadult cuticle and the wrinkled subadult cuticle that has formed beneath it. After the penultimate moult, in the early subadult, the pedipalp tip is inflated and the bulb primordium is located near the distal tip on the ventral side. It is still surrounded by hypodermal tissue, but most of the hypodermis is now associated with the inflated cuticle and shows mesenchymal rather than epithelial organisation. Later during the subadult stage the cavity that has emerged after the penultimate moult gradually fills with coagulated haemolymph material and filaments.

### Differentiation of the sclerites, cymbium and tibia

**Figure 7:**
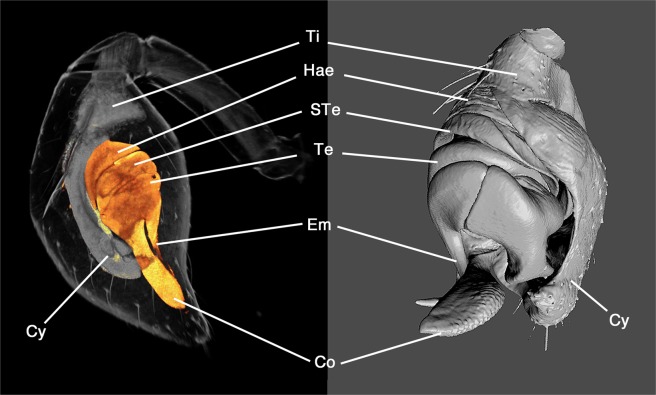
All principal components of the bulb are present at the late subadult stage. Left: Pedipalp club at the late subadult stage, CLSM scan. The bulb primordium is denoted in orange. Right: Adult pedipalp tip with bulb sclerites, 3D reconstruction of a CLSM surface scan. Lines connect corresponding structures. Abbreviations: Co, conductor; Cy, cymbium; Em, embolus; Hae, haematodocha; STe, Subtegulum; Te, tegulum; Ti, tibia.

The hypodermis and the bulb primordium differentiate further during the subadult stage. The most significant change is the compaction of all components by which they attain a shape that comes very close to the final shape of the tarsal tip in the adult, and that detaches the entire tissue from the inflated cuticle of the club (Fig. 4d). The compaction of all components is seen best by the changes in position of important landmarks in the developing pedipalp (please compare coloured landmarks in Fig. 4c,d). In the early bulb primordium (before and shortly after the penultimate moult) no specific substructuring is evident that could be attributed to the forming sclerites. The primordium is oval and has some internal structure, but no outgrowths are yet evident. Later during the subadult stage a small lateral bulge indicates the origin of the embolus (Fig. 4c, orange spot) and the elongated tip of the bulb primordium indicates the growing conductor (Fig. 4c, yellow spot). Both the conductor and the embolus elongate further, but the embolus outgrowth rotates and finally attaches to the outgrowth of the conductor (Fig. 4d, orange spot and yellow spot next to each other). The tibia has a triangular shape from the start, but after compaction this segment is more defined than before (compare purple and green spots in Fig. 4c,d). The cymbium retains a scoop-like shape (see blue spots in Fig. 4c,d which mark the tip of the developing cymbium), but a dramatic change is seen on the opposite side: the ventral portion of the hypodermis is initially similar in shape to the cymbium, but then retracts almost completely from the ventral side (compare the red spot in Fig. 4c,d). At this late subadult stage, a comparison of the structures within the club with the components of the adult male pedipalp shows that all principal components of the adult male pedipalp are already present and have attained almost their final size, shape and correlation within the entire structure (Fig. 7).

### A staging scheme for the developing male copulatory organ

**Figure 8:**
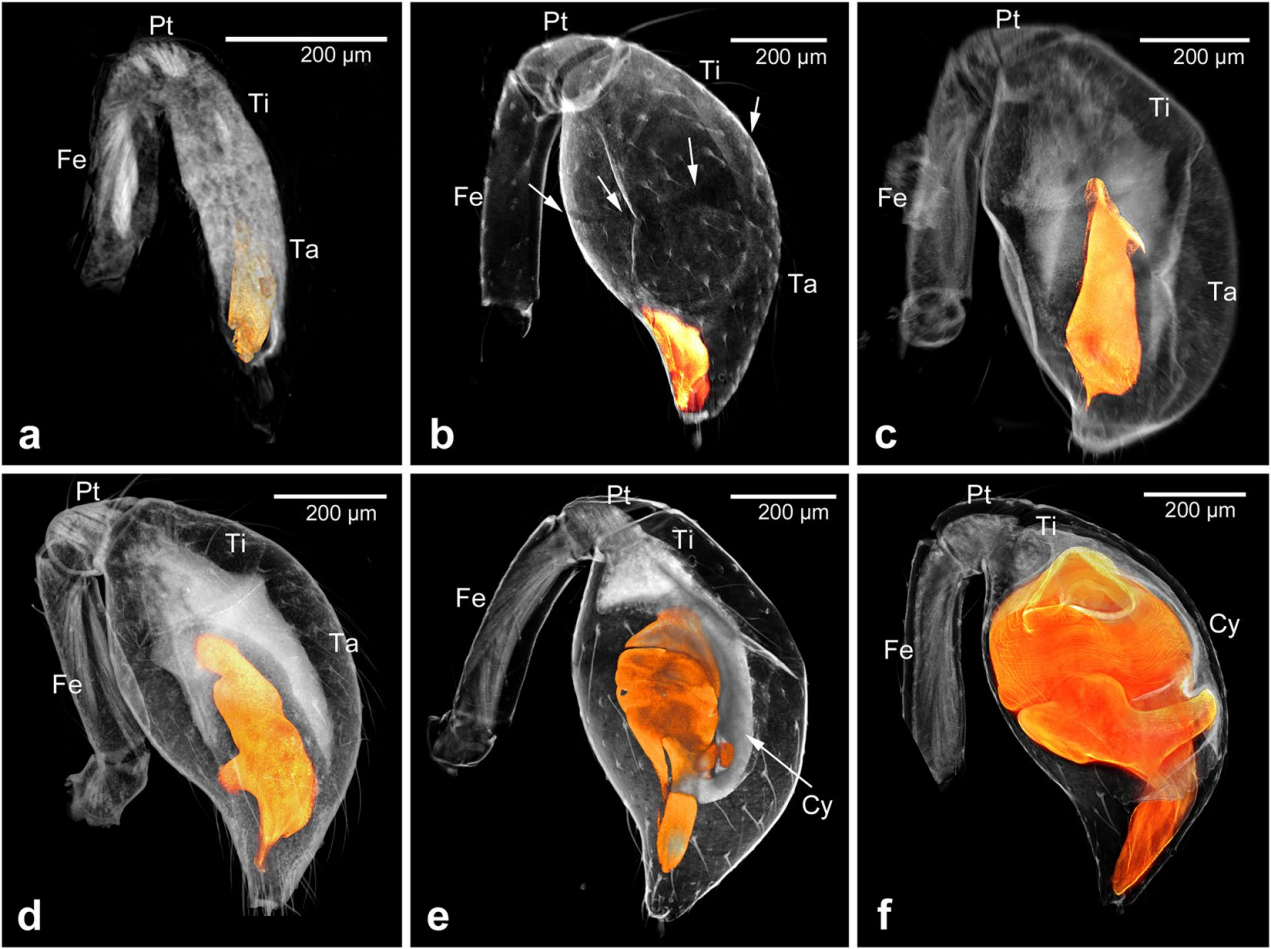
A staging scheme for the developing male copulatory organ. Confocal LSM scans; the bulb primordium is highlighted in orange in all panels. (**a**) Pre-subadult stage. (**b**) Early subadult stage. The arrows point to the remnant of the tibiotarsal joint in the inflated cuticle. (**c**,**d**) Successive mid subadult stage pedipalps. (**e**) Late subadult stage. (**f**) Subadult shortly before the ultimate moult. Scale bars: 200 µm in all panels. Abbreviations: Cy, cymbium; Fe, femur; Pt, patella; Ta, tarsus; Ti, tibia.

In summary, the formation of the bulb starts towards the end of the pre-subadult stage (Fig. 8a). At this stage, the bulb primordium is a small ovoid organ inside the tip of the pedipalp appendage. The penultimate moult leads to a dramatic inflation of the tarsus and tibia, thus leading to the formation of the club, but does not produce major changes in the size or shape of the bulb primordium (Fig. 8b). During the subadult stage the future tibia and cymbium (i.e. the dorsal portion of the tarsus) approach their final size and shape by compaction of the tissue (Fig. 8c–e). The bulb primordium retracts from the location at the tip, and grows in size (Fig. 8c). Distally and at the ventral side, protrusions form that indicate the primordia of the sclerites conductor and embolus (Fig. 8d). The embolus primordium rotates so that it finally attaches to the conductor primordium, both now pointing distally (Fig. 8e). The cymbium has attained the scoop-like shape and the bulb primordium is now lying in this concave scoop, being attached to it via a stalk near the junction between tibia and cymbium. And also the tibia is more compact at this stage, an indication that it has begun attaining its typical bell-cup shape in the adult pedipalp. At this time point all major components and sclerite primordia of the bulb are formed and bulb, tibia and cymbium are maximally compacted and removed from the inflated cuticle of the club. Interestingly, although the entire ensemble of bulb, cymbium and tibia is constantly compacting during development, the outer shape of the club cuticle is not changed. Thus, the size and shape of the outside of the club does not mirror the developmental processes that are taking place inside of the club. In the final phase of the subadult stage, shortly before the ultimate moult, the components of the bulb grow significantly until they fill the entire club (Fig. 8f).

## Discussion

The morphology of the copulatory bulb of the male spider pedipalp is essential for the correct fitting inside the female genital opening and thus for the safe transfer of the sperm. Although bulb morphology is thus important for species recognition and prezygotic reproductive isolation in spiders, the mechanisms that control the development and evolutionary diversification of this structure are virtually unknown. The postembryonic development of the male pedipalp including the male copulatory bulb has been studied only in a few spider species so far, and the bulk of these studies dates back to the early 20th century. The first detailed study of the development of the bulb has been performed by Barrows^14^ on several spider species with a focus on *Steatoda borealis*, a species that belongs to the Theridiidae and is thus closely related to *P. tepidariorum*^15,16^. This author argued that the entire bulb develops from the cells that form the claw fundament in females and juvenile males. Barrows argued that the small group of claw fundament cells undergoes hypertrophy in subadult males thus growing rapidly in size forming the soft portion of the bulb. Because the claw fundament cells are able to segregate sclerotized structures (i.e. the claw), he assumed that the bulb (being a hypertrophied claw fundament) can also segregate sclerotized structures that will form the bulb sclerites (conductor, embolus etc.). Harm^17^ arrives at a similar conclusion, because she finds (in her study on *Segestria bavarica*) that the sclerotized tip of the bulb of this species (the “stylus”) apparently forms at the place where the claw would normally form. However, she stresses that *Segestria bavarica* has a simple bulb, without haematodochae and complex sclerites, and that its morphogenesis might not be representative of spiders with a more complex bulb with several sclerites. Harm^18^ has later studied the development of the bulb of *Evarcha arcuata* (under its synonym *Evarcha marcgravii*) (a jumping spider of the family Salticidae), a species with a more complex bulb. She has confirmed her earlier finding that the distal sclerotized tip of the bulb (the “stylus”) appears to be homologous to the claw. However, she cannot identify the primordia of the remaining sclerites in her sectioned material and their origin and homology therefore remains unclear. A separate study was performed by Gassmann^19^ on the linyphiid spider *Megalepthyphantes nebulosus* (under its synonym *Lepthyphantes nebulosus*), a species with an extremely complex bulb. In Gassmann’s work, the bulb is also formed at the tip of the pedipalp, i.e. near the claw fundament, but he claims no relationship between claw fundament and bulb. Instead, the primordium of the bulb gradually differentiates and develops the future sclerites as new outgrowths entirely unrelated to the claw. In addition, while Barrows and Harm describe the development of the cymbium from the wall of the tarsus, Gassmann writes that the cymbium develops de novo from “organogenic tissue” that fills the pedipalp tip, and later fuses with the differentiated bulb primordium. Bhatnagar and Rempel^20^ have studied another theridiid spider, the black widow *Latrodectus curacaviensis*. They support the conclusions by Barrows and Harm that the bulb develops from a hypertrophied claw fundament, however, their sectioned material shows that the sclerites apparently do not develop from the site of the claw, but appear to be new evaginations of the bulb. These contradictory findings could also not be resolved in two subsequent studies^21,22^. In these works, the location of the claw even changed from the tip of the bulb to the tip of the cymbium, suggesting that the sclerites of the bulb are not homologous to the claw.

Our study of the formation and further development of the bulb in *P. tepidariorum* strongly supports the initial findings by Barrows^14^ and Harm^17,18^. The bulb primordium forms at a location directly beneath the base of the claw of the subadult cuticle, i.e. the primordium apparently comprises (at least partially) those cells that have previously segregated the subadult claw. The primordium then forms an internalised ovoid organ, that subsequently grows and differentiates the primordia of the bulb sclerites as outgrowths from the main body of the organ. This is support for the notion that the bulb sclerites are not an evolutionary novelty of spiders, but instead represent the extremely modified claw of the male adult pedipalp. This makes the bulb sclerites in spiders a unique system to study the mechanisms of evolutionary diversification of a highly modified organ in one sex, and at the same time compare them with the developmental ground-state of the organ (=claw) and its developmental mechanisms which is conserved in the other sex.

Intriguingly, our data show that the strong swelling of the pedipalp in the subadult stage is not caused by the growth of the primordium inside, as previously thought. Instead, the expansion of the cuticle to facilitate the formation of the club is already established at the pre-subadult stage, and the cuticle is then inflated by the influx of haemolymph that rapidly coagulates. The coagulated material, however, does not precipitate irregularly, but it forms a defined structure comprising a central mass and outer filaments. This structure appears to provide a stable framework for the developing bulb, and also serves as a fundament for the morphogenesis of the tibia and the tarsus, because it is partially lined with epithelia and also tethers epithelial connections. Coagulation is a typical physiological response to tissue injury or large scale tissue degeneration (histolysis)^23^. Thus, coagulation in the pedipalp indicates the presence of histolytic processes that transform the tibia-tarsus morphology of the immature male pedipalp into the derived tibia-cymbium morphology of the adult male, similar to histolytic processes during metamorphosis in holometabolous insects^24^. In addition, the coagulated material in the male pedipalp contains inclusions of cell groups, that very likely represent the cell source for the re-establishment of the cymbium and the tibia after histolysis, comparable to the histoblasts and histoblast nests during insect metamorphosis. Notably, the morphology of the coagulated mass is virtually constant in all specimens investigated so far, and therefore its formation seems to be the result of a controlled process, rather than irregular precipitation of haemolymph constituents. In fact, the shape of the coagulated mass prefigures the shape of the cymbium and may therefore function as a blueprint for the histoblasts in building the cymbium proper. This indicates that the coagulated mass in the male pedipalp is not merely a general response to histolysis, and thus suggests a unique role for controlled coagulation after histolysis in the metamorphosis-like morphogenesis of the male pedipalp.

In summary, our study provides a basis for subsequent studies of bulb development in additional spider species. Comparative studies of bulb formation and sclerite specification will provide insight into the homology and evolution of these structures between diverse spider groups, and will also help to link the evolutionary diversification of morphogenetic developmental processes with speciation mechanisms in spiders.

## Materials and Methods

### *Parasteatoda tepidariorum* husbandry

Our *P. tepidariorum* husbandry is kept at controlled temperature (25 °C) and dark/light cycle (10 hours of light). The animals are kept separate in plastic vials sealed with styrene foam plugs and are supplied regularly with water and food. Juveniles are fed with *Drosophila melanogaster* flies, older stages and adults are fed with larger flies (*Musca domestica* and *Lucilia caesar*) or juvenile crickets (*Acheta domesticus*). Water is provided by humid soil.

### Specimen fixation

We have used 27 individuals for study by confocal laser scanning microscopy (CLSM) and diffusible iodine-based contrast-enhanced micro computed tomography (dice-µCT). These spiders were anaesthetised at −20 °C for 7–10 minutes. Then the opisthosoma was removed and only the prosoma was placed in Karlsson and Schultz phosphate buffer (13 mM sodium dihydrogen phosphate monohydrate, 85 mM di-sodium hydrogen phosphate dehydrate, 85 mmol NaCl, 2,5% glutaraldehyde, 4% formaldehyde in water) at 4 °C over night. After fixation, the pedipalps were separated from the spider and the left one was used for CLSM imaging, and the right one was used for dice-µCT imaging. Treatment for CLSM involved bleaching, nucleic acid staining, dehydration and clearing, treatment for dice-µCT involved dehydration, iodine staining and critical point drying (see below).

### Bleaching

After fixation, the pedipalps were placed in 15% hydrogen peroxide in PBS-T and were carefully centrifuged to remove gas bubbles from the tissue. The hydrogen peroxide denatures all pigments. After 1 to 2 days, or longer, when there was no O_2_ development anymore and the pedipalps were not entirely bleached, the pedipalps were transferred to 30% hydrogen peroxide in PBS-T. When the samples were fully bleached they were washed twice with PBS-T.

### Nucleic acid staining

4′,6-diamidino-2-phenylindole (DAPI) was used for DNA detection, because the wavelength of the fluorescence of DAPI proved to be more different from the wavelength of the autofluorescence of the samples. The staining was performed by transferring the pedipalps into a vial with DAPI (1:1000) for 3 h at room temperature in the dark. DAPI was then removed by several washes in PBS-T.

### Dehydration and clearing for CLSM

To dehydrate the pedipalps an ethanol series was performed comprising the following steps for at least 30 min each: 30%, 50%, 70%, 80%, 90%, and 95% ethanol in water. Then the pedipalps were incubated twice in 100% ethanol for half an hour each. After dehydration the ethanol was replaced with 100% methyl salicylate for tissue clearing. Clearing of the pedipalps has been performed as described previously^25^ and on the perforated anodised aluminium slide described recently^26^.

### Confocal laser scanning microscopy (CLSM)

For CLSM scanning a Zeiss LSM 510 microscope was used. An Argon laser with 488 nm wavelength for excitation and a longpass filter for 505 nm wavelength was used for the emitted light. The pinhole was set to 0.81–1.0 airy units. The scan size was set to 1024 × 1024 pixels. The scanning speed was set to 6.4 µs per pixel, line averaging was set to 4. The objectives used were a Zeiss Plan-Neofluar 10x air with a numerical aperture of 0.3 for the whole pedipalp specimen, and a Zeiss Plan-Neofluar 20x air with a numerical aperture of 0.5 to document details.

### Dehydration and iodine staining for dice-µCT

An ethanol series was performed comprising the following steps for at least 30 min each: 30%, 50%, 70%, 80%, 90%, and 95% ethanol in water. Then the pedipalps were incubated twice in 100% ethanol for half an hour each. After the dehydration was complete the pedipalps were stained with iodine (in ethanol) to increase contrast. Iodine changes the interaction of the specimen with the x-rays, through an increase of phase shift and absorption. A solution of 1% iodine in ethanol was added to the vial with fully dehydrated samples overnight, then the samples were rinsed three times with 100% ethanol to remove excess iodine.

### Critical point drying

An automatic critical point dryer (Leica EM CPD300) was used to perform critical point drying. After the samples were dehydrated and iodine stained, they were transferred into a microporous container to avoid losing it due to its very small size. These containers were placed into a larger container filled with 100% ethanol and placed in the critical point dryer. 18 cycles of ethanol/liquid carbon dioxide (CO_2_) exchange were performed to quantitatively remove the ethanol. The CO_2_ was then slowly heated to 31 °C with a pressure of 74 bar. It is essential to perform this phase transfer of CO_2_ very slowly in order to avoid capillary forces or volume changes that would damage delicate morphological structures inside the forming pedipalps.

### Diffusible iodine-based contrast-enhanced micro computed tomography (dice-µCT)

To achieve proper resolution of the soft tissue portions of the samples a self-commissioned laboratory-based X-ray phase-contrast tomography setup was used for dice-µCT imaging^27^. To reach a high resolution of approximately 1 µm despite a relatively high focal spot size of the X-ray source (70 µm) an inverse geometry (source-to sample distance » sample-to-detector distance) in combination with the high resolution detector XSight (Rigaku, Prague, Czech Republic) was used. The resolution of this setup is limited to the detector point spread function (0.54 µm) due to negligible optical magnification. The detector´s field of view is 1.8 × 1.4 mm, but we chose as the maximum sample dimensions 0.8 × 0.5 × 0.5 mm, because this makes it much easier to perform tilt corrections in the reconstruction step. A detailed setup description has been published previously^27^. For tomographic reconstruction 25 dark-field images, 25 flat-field images and 1000 projections over 180° were recorded with an exposure time of t = 40 s each. To increase the signal to noise ratio, each empty-beam corrected projection was binned by a factor of 2. To retrieve the phase information the Bronnikov-Aided-Correction algorithm (BAC) was applied on each projection^28,29^. The tomographic reconstruction was performed with the (cone-beam) filtered back-projection implementation of the ASTRA toolbox^30–32^.

### Image segmentation and processing

dice-µCT and CLSM 3D-stacks were processed in Amira 5.4.1 (FEI SAS, France, www.vsg3d.com). Structures of interest were marked with the brush or magic wand tool in the segment editor. With the brush tool individual or groups of pixels are marked by the user, and the magic wand is a grey value based region growing algorithm, where the user sets the seeding points and is able to set limit lines to define borders for the growth. To highlight them, the volren-module was connected to the image stack and label field, respectively, then in the port transfer functions two colormaps gray.am and glowred.col were set up with the AlphaScale in gray.am tuned to 20–30%. To assign the glowred.col colormap to the structure of interest the desired colormap was chosen in the material port by selecting the colormap icon. To insert scale bars the module scale was activated and for correct distances the orthographic camera was used in the viewer window. Images for figures were taken with the snapshot tool. Images were then corrected for contrast and colour values and combined into figures using Adobe Photoshop CS5 for Apple Macintosh.

## Supplementary information

Supplementary Information

Supplementary Table S1

Supplementary Table S2

## Data Availability

The datasets generated and analysed during the current study are available from the corresponding author on reasonable request.
