# Supplementary material for: Formation and development of the male copulatory organ in the spider Parasteatoda tepidariorum involves a metamorphosis-like process
Source: Sci Rep. 2019 May 6;9:6945. doi: 10.1038/s41598-019-43192-9 (PMC6502807; doi:10.1038/s41598-019-43192-9)
Supplement: Supplementary file 1 — Supplementary Information [file 41598_2019_43192_MOESM1_ESM.pdf]

Supplementary Information

Formation and development of the male copulatory organ in the spider *Parasteatoda tepidariorum* involves a metamorphosis-like process

Felix Simon Christian Quade, Jana Holtzheimer, Jasper Frohn, Mareike Töpperwien, Tim Salditt, Nikola-Michael Prpic

Supplementary Table S1

(XLSX-File, 11 kB) Overview of all monitored individuals (n=55). Column B gives the recorded date of the penultimate moult and column C gives the date (if recorded) for the ultimate moult. Column D gives the sex of the individual as confirmed after the penultimate moult, while column E gives the sex that was assigned to the specimen on the basis of the sexing at the pre-subadult stage. In most cases this assumed sex was later confirmed. Column F gives the duration (in days) of the subadult stage for all males for which both the penultimate and the ultimate moult have been recorded.

Supplementary Table S2

(XLSX-File, 9 kB) Overview of the feeding and watering protocol during the observation of the penultimate and ultimate moults. The animals were monitored from November 2nd, 2016 to December 5th, 2016 and were regularly supplied with water (column B) and food (column C). They were also regularly examined for moulting (column D).

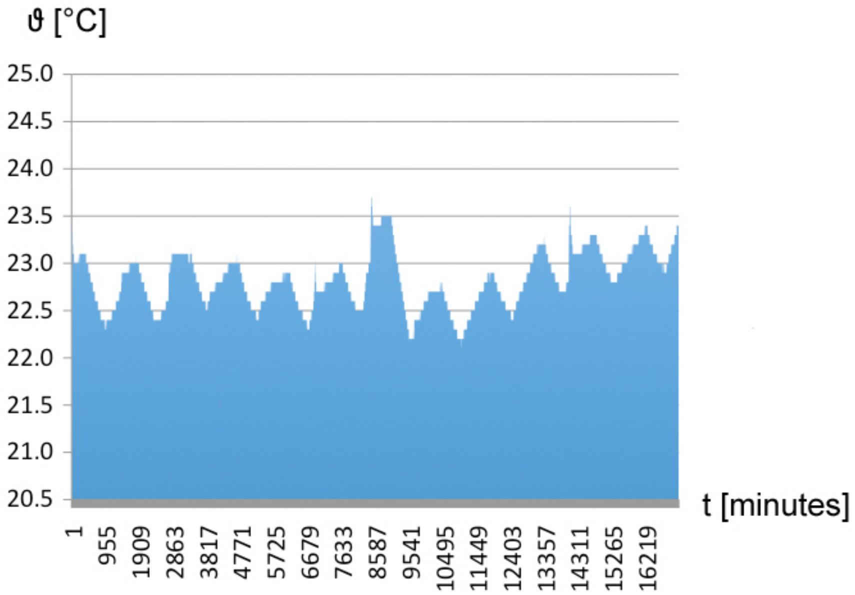

Supplementary Figure S1

Temperature log of the incubator used for the moulting observations. The x-axis gives the time measured in minutes. The y-axis gives the temperature measured in °C. The incubator showed a slight circadian oscillation.

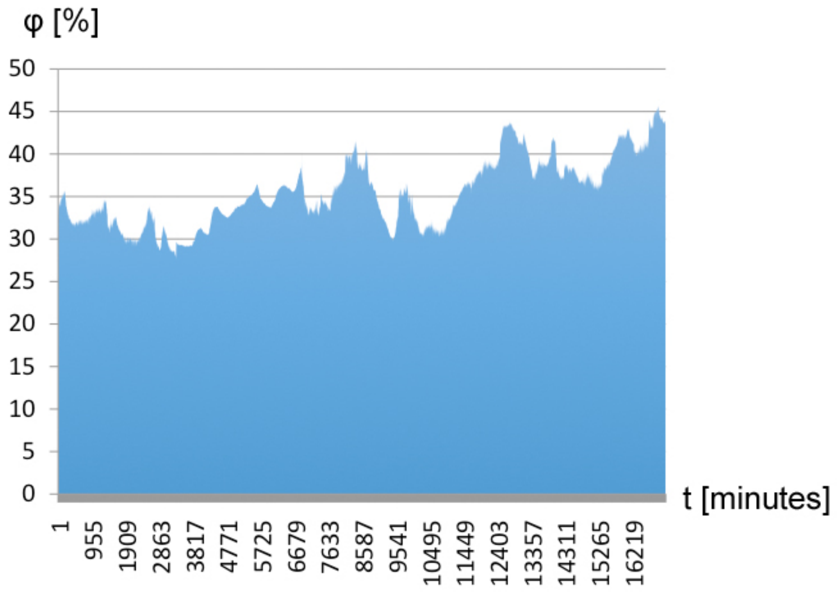

Supplementary Figure S2

Relative humidity log of the incubator used for the moulting observations. The x-axis gives the time measured in minutes. The y-axis the relative humidity given in per cent. The incubator shows an irregular undulation of relative humidity.
